# An Unwanted Guest in the Biliary Tract: A Case Report

**DOI:** 10.31729/jnma.4612

**Published:** 2019-10-31

**Authors:** Nita Lohala, Ram Bahadur Gurung, Nishan Bhattarai, Anjila Lama

**Affiliations:** 1 Kathmandu University School of Medical Sciences, Dhulikhel, Kavre, Nepal; 2 Department of Internal Medicine, Dhulikhel Hospital, Dhulikhel, Kavre, Nepal

**Keywords:** *ascariasis*, *ascaris lumbricoides*, *cholangiopancreatography endoscopic retrograde*, *common bile duct.*

## Abstract

Ascariasis is a frequent human gastrointestinal tract helminthic disease caused by Ascaris lumbricoide. It usually stays in the intestinal lumen and occasionally migrates into the biliary tract through ampulla of Vater. Biliary ascariasis is a critical complication of intestinal ascariasis with life-threatening manifestations. We report a case of a-38 year-old lady who presented with colicky type epigastric pain radiating to back with diffuse tenderness over abdomen on examination. Ultrasonography abdomen showed linear echogenic structure in common bile duct. Biliary ascariasis was noted on Endoscopic Retrograde Cholangio Pancreaticography following which extraction was done. Our report highlights the varied clinical features of biliary ascariasis.

## INTRODUCTION

Ascariasis is a frequent helminthic disease of gastrointestinal tract in human caused by a parasitic roundworm Ascaris lumbricoides. It mostly manifests in tropical developing countries. The adult form of A. lumbricoides usually stays in the intestinal lumen in the jejunum. Occasionally, the adult worm can migrate into the biliary tract through the ampulla of Vater when there is increased parasite load and cause choledocholithiasis-like symptoms.^1^

Biliary ascariasis is a critical complication of intestinal ascariasis with life-threatening manifestations.^2^ The diagnosis of biliary ascariasis is difficult.^3^ Our report highlights the varied clinical features of biliary ascariasis.

## CASE REPORT

A 38-year-old lady presented with complaints of epigastric pain for 5 day which was sudden in onset, colicky and lasted for 5 to 10 minutes. It radiated to back and there were no aggravating and relieving factors. There is no history of vomiting, abdominal distension, waterbrash, nausea, chest pain, fever, and headache. Bladder and bowel habit was normal. She didn’t smoke and didn’t consume alcohol. There is no history of chronic illness in the past. General examination was normal and vitals were within the normal range. Guarding was present with diffuse tenderness over the abdomen. Tympanic note was heard on percussion and bowel sounds were heard. Other systemic examinations were normal.

**Figure 1:**
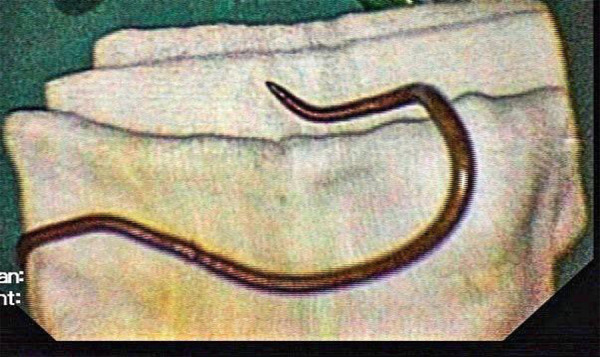
Single roundworm extracted from the common bile duct.

Relevant investigations were sent. Pancreatic enzymes were within the normal range (Amylase- 58, Lipase-34). Blood counts were within the normal range (Total Lymphocyte count- 10600, Neutrophils- 57%, Lymphocytes- 20%). Hemoglobin was 15.4 gm/dl and platelets were 256000. Liver function test showed total bilirubin of 0.9, direct bilirubin of 0.4, ALT of 56, AST of 43, ALP of 67, Total protein of 6.6 and albumin of 4.4. Urine routine and microscopic examination was normal and the urine pregnancy test was negative. Ultrasonography abdomen showed atrophic left liver with minimal thickening of the intrahepatic common bile duct (CBD) and minimal intrahepatic biliary radicals dilatation. Linear echogenic structure was noted in CBD. Endoscopic Retrograde Cholangio-Pancreaticography was done which showed biliary ascariasis and the single worm was extracted from CBD (Figure 1).

She was treated with intravenous medications (Injection Pantoprazole, Injection Tramadol, and Ondansetron) for symptomatic treatment and deworming was done with Albendazole 400 mg once a day dose for 3 days. Her condition gradually improved and epigastric pain gradually subsided.

## DISCUSSION

The disease may stay asymptomatic with ascarids in the lumen of small intestine. Symptoms can occur when helminths invade the biliary or pancreatic ducts. Clinical signs depend on where the worm migrates: if it reaches the gallbladder—cholecystitis, and if it travels to the bile duct or pancreatic duct—cholangitis or pancreatitis, respectively, can be seen.^1^ Although many hypotheses have been formed to explain the migration of A. lumbricoides to various organs, an important prerequisite for their reaching the biliary tract seems to be a heavy infection.^4^ Its presence can lead to a multitude of presentations, one of the rarer ones being obstructive jaundice due to migration of the worm into the biliary tree.^3^ Previous studies have shown that almost 30% of patients with biliary ascariasis have a prior history of cholecystectomy.^5^ However, in our case, there was no jaundice and history of previous cholecystectomy. The only clinical presentation was colicky type of epigastric pain.

As biliary ascariasis does not have any characteristic laboratory or clinical features, radiologic imaging methods play an important role in the diagnosis of a parasite in the biliary tree.5 Recognition of this disease is facilitated by the liberal usage of real-time sonography and ERCP.^6^ ERCP is a diagnostic as well as a treatment procedure.^1^ ERCP combined with antihelminthic agents is the first-line treatment of biliary ascariasis.^7^ In our case, the intervention was done as proposed in the medical literature. Invasive methods were essential because of suspected biliary obstruction.

Ascariasis is recognized as a major health problem in endemic regions of the world so clinicians need to be aware of its complications like hepatobiliary and pancreatic ascariasis with unusual presentation and laboratory findings.^8^

Consent: JNMA Case Report Consent Form was signed by the patient and the original article is attached with the patient’s chart.

## Conflict of Interest:

None.
